# Investigating the Binding Heterogeneity of Trace Metal Cations With SiO_2_ Nanoparticles Using Full Wave Analysis of Stripping Chronopotentiometry at Scanned Deposition Potential

**DOI:** 10.3389/fchem.2020.614574

**Published:** 2020-12-16

**Authors:** Elise Rotureau, Luciana S. Rocha, Danielle Goveia, Nuno G. Alves, José Paulo Pinheiro

**Affiliations:** ^1^Université de Lorraine, CNRS, LIEC, Nancy, France; ^2^Centro Interdisciplina de Quimica do Algarve (CIQA), Departamento de Quimica e Bioquimica (DQB)/Faculdade de Ciencia e Tecnologia (FCT), University of Algarve, Faro, Portugal; ^3^Universidade Estadual Paulista (Unesp)-Campus de Itapeva, Itapeva, Brazil

**Keywords:** trace metal, binding heterogeneity, SiO_2_ nanoparticles, SSCP, AGNES

## Abstract

Silica oxides nano- and microparticles, as well as silica-based materials, are very abundant in nature and industrial processes. Trace metal cation binding with these bulk materials is generally not considered significant in speciation studies in environmental systems. Nonetheless, this might change for nanoparticulate systems as observed in a previous study of Pb(II) with a very small SiO_2_ particle (7.5 nm diameter). Besides, metal binding by those nanoparticles is surprisingly characterized by a heterogeneity that increases with the decrease of metal-to-particle ratio. Therefore, it is interesting to extend this study to investigate different trace metals and the influence of the nanoparticle size on the cation binding heterogeneity. Consequently, the Cd(II), Pb(II), and Zn(II) binding by two different sized SiO_2_ nanoparticles (Ludox LS30 and TM40) in aqueous dispersion was studied for a range of pH and ionic strength conditions, using the combination of the electroanalytical techniques Scanned Stripping ChronoPotentiometry and Absence of Gradients and Nernstian Equilibrium Stripping. The coupling of these techniques provides the free metal concentration in the bulk (AGNES) and information of the free and complex concentration at the electrode surface for each Stripping Chronopotentiometry at Scanned deposition Potential (SSCP). A recent mathematical treatment allows the reconstruction of a portion of the metal to ligand binding isotherm with the included heterogeneity information using the full SSCP wave analysis. In this work, we observed that the Zn(II) binding is homogeneous, Cd(II) is slightly heterogeneous, and Pb(II) is moderately heterogeneous, whereas the results obtained with the 7.5 nm diameter nanoparticle are slightly more heterogeneous than those obtained with the one of 17 nm. These findings suggest that the Zn(II) binding is electrostatic in nature, and for both Cd(II) and Pb(II), there should be a significant chemical binding contribution.

## Introduction

The mobility and the bioavailability of trace metal elements (TME) in aquatic systems are largely mediated by their interaction with organic ligands, such as humic matter, exopolymeric substances, and/or inorganic surfaces, such as clays, silicates, aluminum, iron, and manganese (hydro)oxides (Buffle, 1988; Lead and Wilkinson, 2006).

Physicochemical heterogeneity is an intrinsic characteristic of most of these natural particles (Riemsdijk and Koopal, 1992; Duval et al., 2005; Duval and Gaboriaud, 2010), which generally arises from the diverse structures and morphologies of the particles, their polyfunctional chemical binding sites, and the polyelectrolytic nature of the particle charge. Accordingly, the metal ions binding/adsorption toward these natural particles is chemically heterogeneous, with binding association or adsorption constants that increase with the decrease of the metal-to-ligand ratio. This heterogeneity was initially estimated using the empirical Γ parameter of a Freundlich-type isotherm, which averages all contributions, covalent and electrostatic into a single descriptor (Filella and Town, 2001).

Later, the modeling of metal ion adsorption in mineral surfaces was refined by the separation of electrostatic and chemical binding contributions within the framework of surface complexation models [Dzombak and Morel (1990), CD-Music (Hiemstra and Van Riemsdijk, 1996)]. Similarly, the modeling of metal ion binding by natural organic matter was improved by the introduction of complexation codes that consider both an electrostatic contribution and a chemically heterogeneous metal ion binding by either a sum of discrete sites WHAM (Tipping, 1994) or a continuous site distribution (NICA) (Kinniburgh et al., 1996; Milne et al., 2003).

The experimental study of chemical heterogeneity in natural systems is complicated due to the low levels of trace metals and the complex interplay between those and the different ligands in solution. Recently, we proposed a new methodology in Pinheiro et al. (2020a) to investigate trace metal binding heterogeneity based on the full wave analysis of the electroanalytic technique of Stripping Chronopotentiometry at Scanned deposition Potential (SSCP). From the conversion of the experimental data obtained at the electrode surface, it is possible to recover a portion of the binding isotherm at realistic environmental trace metal concentrations.

The objective of this work is to apply this novel methodology to study the surprising metal binding heterogeneity of silica nanoparticles, first reported for Pb(II) ions by Goveia et al. (2011). The origin of this phenomenon remains uncertain, since at first sight one did not expect a significant chemical heterogeneity from metal binding to amorphous SiO_2_ nanoparticles considering their chemically homogeneous composition and monodisperse particle size distribution. Several electrokinetic (Allison, 2009) or aggregation (Škvarla and Škvarla, 2017) studies reported a gel-like layer at the silica/solution interphase, namely, a gradual distribution of the component material from the particle core to the particle/water interphase. For charged particles, the presence of a water- and ion-permeable surface layer implies a tridimensional distribution of functional sites. An electric field spans from the bulk electrolyte solution to the inner part of the particle, likely generating a spatially dependent metal complexation heterogeneity. Several types of chemical metal complexes may be formed at the particulate interfaces, such as the monodentate or bidentate complexes that may occur at the orthosilicic acid sites in the SiO_2_ nanoparticle. Additionally, structural impurities occluding the silica surface may arise from the synthesis of silica, which are depicted, e.g., by the isomorphic substitution of one Si by one Al atom (Bergna and Roberts, 2005).

In this work, we applied a methodology consisting first in a qualitative diagnosis of the effects of heterogeneity on the SSCP wave (Town and van Leeuwen, 2004; Town, 2008; Rotureau, 2014; Rotureau et al., 2016) according to (i) the nature of the metal ions, where we used Pb(II), Cd(II), and Zn(II), (ii) the physicochemical conditions of the medium, namely, the pH and ionic strengths of the solution, and (iii) the variation of particles size, using nanoparticles of 8 and 17 nm radius. Second, the quantitative reconstruction of the low coverage fraction of the binding isotherm contained in the SSCP wave is achieved using the mathematical treatment developed by Pinheiro et al. (2020a).

## Materials and Methods

### Reagents

The chemicals used in the present work were of analytical reagent grade and used as received, unless stated otherwise. All solutions were prepared with ultra-pure water (18.3 MΩ cm, Milli-Q systems, Millipore-waters). The nitric acid 65% (suprapur) and the standard stock solutions of mercury nitrate (1,001 ± 2 mg L^−1^), cadmium nitrate (999 ± 2 mg L^−1^), lead nitrate (999 ± 2 mg L^−1^), and zinc nitrate (1,000 ± 2 mg L^−1^) were purchased from Merck. Cd(II), Pb(II), and Zn(II) solutions were prepared from dilution of the certified standard. Ludox® TM40 [40% (w/w) suspension in water] and LS30 [30% (w/w) suspension in water] colloidal silica (SiO_2_) were purchased from Aldrich. Sodium nitrate electrolyte solution (10, 30, and 100 mM), MES [2-(N-morpholino)ethanesulfonic acid] buffer (200 mM), and MOPS [3-(N-morpholino)propanesulfonic acid] buffer (200 mM) were prepared from solids (Merck, suprapur and Merck >99%, respectively). The pH adjustments were performed using nitric acid (Merck, suprapur) and sodium hydroxide (100 mM standard, Merck) solutions.

Potassium thiocyanate, hydrochloric acid, and potassium chloride, all p.a. from Merck, were used to prepare the solution for the re-dissolution of the mercury film. Solutions of ammonium acetate [NH_4_CH_3_COO (1,000 mM)/CH_3_COOH (1,000 mM)] (Merck) were prepared monthly and used without further purification.

### Electrochemical Apparatus

An Ecochemie Autolab PGSTAT10 and μAutolab potentiostats (controlled by GPES 4.9 software from EcoChemie, the Netherlands) were used in conjunction with a Metrohm 663 VA stand (Metrohm, Switzerland). A three electrode configuration was used comprising a Hg thin film plated onto a rotating glassy carbon (GC) disk (2 mm diameter, Metrohm) as the working electrode, a GC rod counter electrode, and an Ag/AgCl reference electrode from World Precision Instruments DRIREF-5 (electrolyte leakage <8 × 10^−4^ μl h^−1^). A Denver Instrument (model 15) and a Radiometer analytical combined pH electrode calibrated with Titrisol buffers (Merck) were used to measure pH.

### Preparation of the GC Substrate

Prior to deposition of the Hg films, the GC electrode was conditioned following a previously reported polishing/cleaning procedure (Monterroso et al., 2004). In brief, the electrode was polished with alumina slurry (grain size 0.3 μm, Metrohm) and sonicated in pure water for 60 s to obtain a renewed surface. Then, an electrochemical pre-treatment was carried out using a 50× cyclic voltammetric scan between −0.8 and +0.8 V at 0.1 V s^−1^, in NH_4_CH_3_COO (1,000 mM)/HCl (500 mM) solution. The surface area of the GC electrode was measured by chronoamperometry in 1.124 mM ferricyanide/1,000 mM KCl solution (purged for 300 s). Before the measurements, the solution was stirred for 30 s (2,000 rpm) and followed by a resting period of 120 s. The parameters used were: *E* = 0.5 V and *t* = 3 s, and the measured response was the current *I* as a function of time *t*. The electrochemically active area of the GC electrode was calculated from the slope of *I* vs. *t*^−1/2^ Cottrell equation (diffusion coefficient of ferricyanide *D* = 7.63 × 10^−10^ m^2^ s^−1^). The electrochemically active area obtained was (3.334 ± 0.062) × 10^−6^ m^2^ (two polishing experiments, each with four replicate determinations). When not in use, the bare GC electrode was stored dry in a clean atmosphere.

### Preparation of the Hg Electrode

The thin Hg film was prepared *ex-situ* in 0.12 mM Hg(II) nitrate in nitric acid 0.73 mM (pH 1.9) by electrodeposition at −1.3 V for 700 s at a rotation rate of 1,000 rpm. The charge associated with the deposited Hg (*Q*_Hg_) was calculated by electronic integration of the linear sweep stripping peak of Hg, for *v* = 0.005 V s^−1^. The electrolyte solution was ammonium thiocyanate 5 mM (pH 3.4). The stripping step began at −0.15 V and ended at +0.4 V (Rocha et al., 2007).

### SSCP and AGNES-SCP Measurements

Stripping chronopotentiometric measurements were carried out in 20 ml 10, 30, and 100 mM NaNO_3_ solutions containing a concentration of 5.0 × 10^−4^ mM of the following individual metals: Cd(II), Pb(II), and Zn(II). The experimental conditions used were: deposition time (*t*_d_) 45 s, oxidizing current (*I*_s_) 2 × 10^−6^ A, applied until the potential reached −0.30 V, and electrode rotation speed 1,000 rpm. All solutions were purged for 20 min at the beginning of every experiment and for 20 s (assisted by mechanical stirring of the rotating electrode) after each stripping chronopotentiometry (SCP) measurement. Measurements were made for a range of deposition potentials, from the foot to the plateau of the SSCP wave, i.e., from −1.25 to −1.00 for Zn(II), from 0.85 to −0.60 V for Cd(II), and from −0.70 to −0.40 V for Pb(II). The free metal ion concentration was determined by the AGNES-SCP according to the procedure developed by Parat et al. (2011). The measurements were performed by applying a deposition potential *E*_1_ of −1.085, −0.655, and −0.465 V, for Zn(II), Cd(II), and Pb(II), respectively, and for a period of time *t*_1_ ranging between 240 and 300 s. All measurements were carried out at room temperature (21–23°C).

## Theory

The theoretical basis for SCP and its use in SSCP are well-established in the literature. Therefore, the reader is referred to the supporting information and reference (van Leeuwen and Town, 2002) for more details on the theoretical aspects of the technique. The principles and key equations relevant for the present work in brief are evoked here. The SCP measurement comprises two steps. During the first stage, metal ions are accumulated at the electrode by application of a constant deposition potential (*E*_d_) for a given time (*t*_d_). Then, the metal is re-solubilized by imposing a constant oxidizing current (*I*_s_), and the resulting analytical time (τ) is directly proportional to the quantity of total accumulated metal. SSCP waves are obtained from a series of SCP measurements achieved at different *E*_d_ and by plotting the data couple (τ, *E*_d_).

Considering the formation of a labile 1:1 metal complex, ML, between the electroactive metal ion M and the ligand L:

(1) $$\begin{array}{l} \text{M}+\text{L}\overset{k_{\text{d}}}{\underset{k_{\text{a}}}{⇄}}\text{ML}\\ ↓↑\;\;n{\text{e}}^{-}\\ {\text{M}}^{0} \end{array}$$

where *k*_a_ and *k*_d_ are the association and dissociation rate constants, respectively. The system is dynamic at bulk level if the rates for the volume reactions are fast on the experimental time scale, *t*:

(2) $$\begin{array}{r} k_{\text{d}}t,\;k_{\text{a}}^{\prime }t>>1 \end{array}$$

where $k_{a}^{\prime }=k_{a}c_{L,t}^{*}$. For labile complexes, the rates of dissociation/association are sufficiently high relative to the experimental timescale, to maintain full equilibrium between complexed and free metal (van Leeuwen and Town, 2002; Town and van Leeuwen, 2003). Under conditions of sufficiently excess of ligand (as typically used in stripping experiments), $k_{\text{a}}^{\prime }$ is approximately constant, and we can define $K^{\prime }=\frac{k_{a}^{\prime }}{k_{d}^{\prime }}=\frac{c_{\text{ML}}^{*}}{c_{\text{M}}^{*}}=Kc_{\text{L},\text{T}}^{*}$, where *K* represents the stability constant of ML, and $c_{\text{L},\text{T}}^{*}$ represents the total ligand concentration in the bulk solution (mM).

### For Homogeneous and Labile Metal Binding Systems

For a given potential, the deposition current, $I_{d,\text{M}+\text{L}}^{*}$, originating from reduction of the metal ion of interest is given by:

(3) $$\begin{array}{r} I_{d,\text{M}+\text{L}}^{*}=\frac{nFA\bar{D}c_{\text{M},\text{T}}^{*}}{\bar{\delta }} \end{array}$$

where $c_{\text{M},\text{T}}^{*}$ is the total metal concentration in the bulk solution (mM), *A* is the electrode surface area, $\bar{D}$ is the mean diffusion coefficient of the metal ion (m^2^ s^−1^) given by:

(4) $$\begin{array}{r} \bar{D}=\frac{D_{\text{M}}c_{\text{M}}^{*}+D_{\text{ML}}c_{\text{ML}}^{*}}{c_{\text{M},\text{T}}^{*}} \end{array}$$

and $\bar{\delta }$(m) is the thickness of the diffusion layer, which is expressed by, for a RDE (Levich, 1962):

(5) $$\begin{array}{r} \bar{\delta }=1.61\;{\bar{D}}^{1/3}\omega^{-1/2}\nu^{1/6} \end{array}$$

The characteristic time constant for the deposition process τ_*d*_ (s) for a mercury drop or film electrode is defined by:

(6) $$\begin{array}{r} \tau_{d}=\frac{V_{Hg}\bar{\delta }}{A\bar{D}\left(1+K^{\prime }\right)\theta } \end{array}$$

where θ is the free metal surface concentration ratio for a given deposition potential, and *V*_*Hg*_ (m^3^) is the volume of the mercury electrode.

Then, the equation for the SSCP wave for a fully labile complex ML, τ, is given by:

(7) $$\begin{array}{r} \tau =\frac{I_{\text{d},\text{M}+\text{L}}^{*}\tau_{d}}{I_{\text{S}}}\left[1-\exp\left(\frac{t_{d}}{\tau_{d}}\right)\right] \end{array}$$

Thermodynamic complex stability constants, *K*′, can be retrieved from the shift in the half-wave deposition potential, Δ*E*_*d*, 1/2_ between the SSCP curve with metal only and the one after addition of ligands (DeFord and Hume, 1951):

(8) $$\begin{array}{r} \ln\left(1+K^{\prime }\right)=-\left(\frac{nF}{RT}\right)\Delta E_{d,1/2}-\ln\left(\frac{\tau_{\text{M}+\text{L}}^{*}}{\tau_{\text{M}}^{*}}\right) \end{array}$$

where $\tau_{\text{M}}^{*}$ and $\tau_{\text{M}+\text{L}}^{*}$ are the limiting wave heights in the absence and in the presence of ligands, respectively. *R* is the gas constant, *F* is the Faraday constant, *n* is the number of electrons involved in the reduction/oxidation processes, and *T* is the temperature.

### For Heterogeneous Metal Binding Systems

SSCP is also able to provide an evaluation of the chemical heterogeneity through the changing slope of the SSCP wave. For heterogeneous ligands systems, constructing SSCP curves involves also probing a range of metal complexes with various stability constants along the slope of the wave. At the foot of the wave, the weaker metal complexes dissociate to release the free metal toward the electrode, whereas by going closer to the top of the wave, the stronger ones start to contribute to the electrochemical signal. As a result, the SSCP wave is more elongated regarding the homogeneous case, and the extent of the spreading out of the wave reflects the degree of heterogeneity, as can be observed for the metal/humic acid curves in Pinheiro et al. (2020a).

### Wave Analysis in Heterogeneous Metal Binding Systems and Computation of **c**_*M*_**(0, t**_**d**_**)** and **c**_*ML*_**(0, t**_**d**_**)** at the Electrode Surface

The concentrations *c*_M_(0, *t*_*d*_) and *c*_ML_(0, *t*_*d*_) are determined for every experimental point of the SSCP curves in the region of the slope portion (Pinheiro et al., 2020a).

As detailed in Serrano et al. (2007), the free metal ion concentration at the electrode surface can be evaluated by coupling the amount of reduced metal accumulated in the electrode together with the Nernst equation. Under the conditions where a RDE is used, the equation becomes (Pinheiro et al., 2020a):

(9) $$\begin{array}{r} c_{\text{M}}^{0}=c_{\text{M}}\left(0,t_{\text{d}}\right)=u_{1/2}c_{\text{T},\text{M}}^{*}\frac{\tau }{\tau_{\text{M}}^{*}}\exp\left(\frac{nF}{RT}\left(E_{d}-E_{d,1/2}^{\text{M}}\right)\right) \end{array}$$

where *u*_1/2_ is 1.5936 (Rocha et al., 2015), and $E_{d,1/2}^{\text{M}}$ is the deposition half-wave potential in the system with only metal.

The determination of *c*_*ML*_(0, *t*_d_) arises from the balance of the arriving flux of metal species at the electrode times the electrode area with the time variation of the accumulated number of moles, which gives the following differential equation:

(10) $$\begin{array}{r} c_{ML}^{0}=c_{ML}\left(0,t_{d}\right)=c_{ML}^{*}-\frac{dc_{\text{M}}\left(0,\zeta \right)}{d\zeta }+\frac{c_{\text{M}}^{*}-c_{\text{M}}^{0}}{\varepsilon^{2/3}} \end{array}$$

In this expression, ε corresponds to the coefficient diffusion ratio $\frac{D_{\text{ML}}}{D_{\text{M}}}$, and ζ stands for a new variable:

(11) $$\begin{array}{r} \zeta =\frac{\varepsilon^{2/3}u_{1/2}t}{t_{d}}\;\exp\left(\frac{nF}{RT}\left(E_{d}-E_{d,1/2}\right)\right) \end{array}$$

The total metal concentration at the electrode surface $c_{M,T}^{0}$ is thus given by the sum of $c_{ML}^{0}$ and $c_{M}^{0}$. Along the SSCP wave, the $c_{M,T}^{0}$ varies from the bulk value ($c_{M,T}^{*}$) in the very beginning of the wave to very close to zero in the plateau. Since the total ligand concentration is in excess over the total metal, investigating the individual points along the wave is analogous to performing a bulk metal titration at fixed ligand concentration.

In Equation (10), the bulk free metal ion concentration $c_{\text{M}}^{*}$ is experimentally determined using the AGNES technique for which the theoretical and experimental details are recalled in the supporting information.

## Results

To investigate qualitatively the binding heterogeneity from the SSCP waves, two normalization operations are needed. First, each point of the SSCP wave (τ) is divided by its limiting value (τ^*^), thus obtaining a dimensionless y-axis (τ^norm^) normalizing the differences in transport (diffusion coefficients of the different species) and total metal concentrations. Then, the so-obtained τ^norm^ in the presence of ligand is subtracted point by point from the metal only calibration yielding the potential difference (Δ*E* = *E*_d,tit_–*E*_d,cal_). This procedure normalizes the potential shift regarding the different metal standard potential reductions, thus allowing the comparison of the three metals in the x-axis.

Due to the experimental errors, it is necessary to ignore at least the points below 0.05 and above 0.95 in the y-axis after normalization (Pinheiro et al., 2020a). Nevertheless, it was observed that in some cases, it is required to neglect more points due to the influence of point scattering in the derivative computation (Equation 10).

### Evaluation of the Heterogeneity for the Different Metal Ions

**Figure 1:**
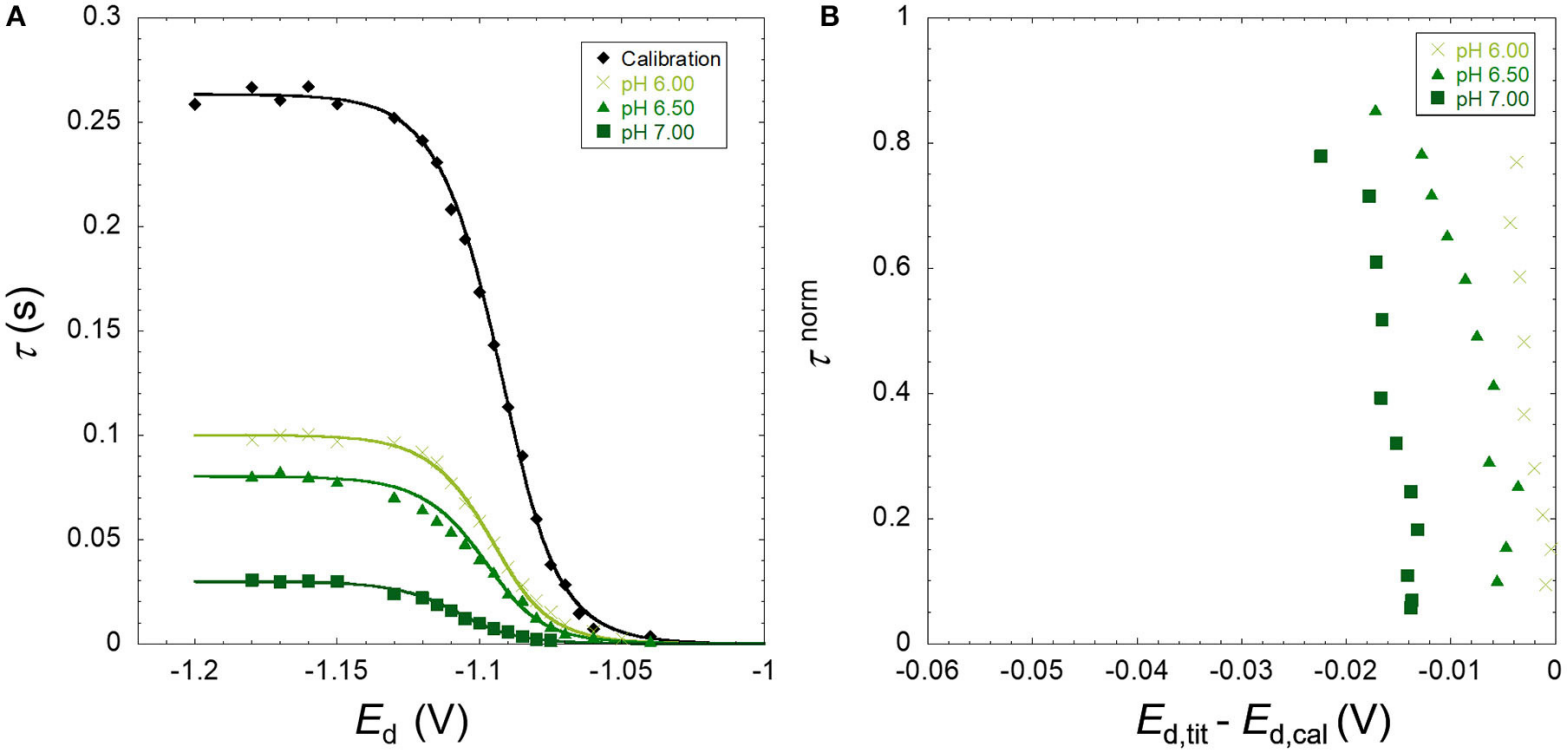
**(A)** Experimental SSCP waves for the zinc only at pH 4 (calibration) and in the presence of SiO_2_ nanoparticles (TM40) obtained at 10 mM NaNO_3_, pH 6.00, 6.50, and 7.00 for a total Zn concentration of 5 × 10^−4^ mM and particle concentration of 0.3 wt%. The lines represent the analytical simulation of the experimental results as described in the theoretical section. **(B)** The normalized analytical times τ^norm^ derived from the SSCP waves plotted against *E*_d,tit_–*E*_d,cal_.

**Figure 2:**
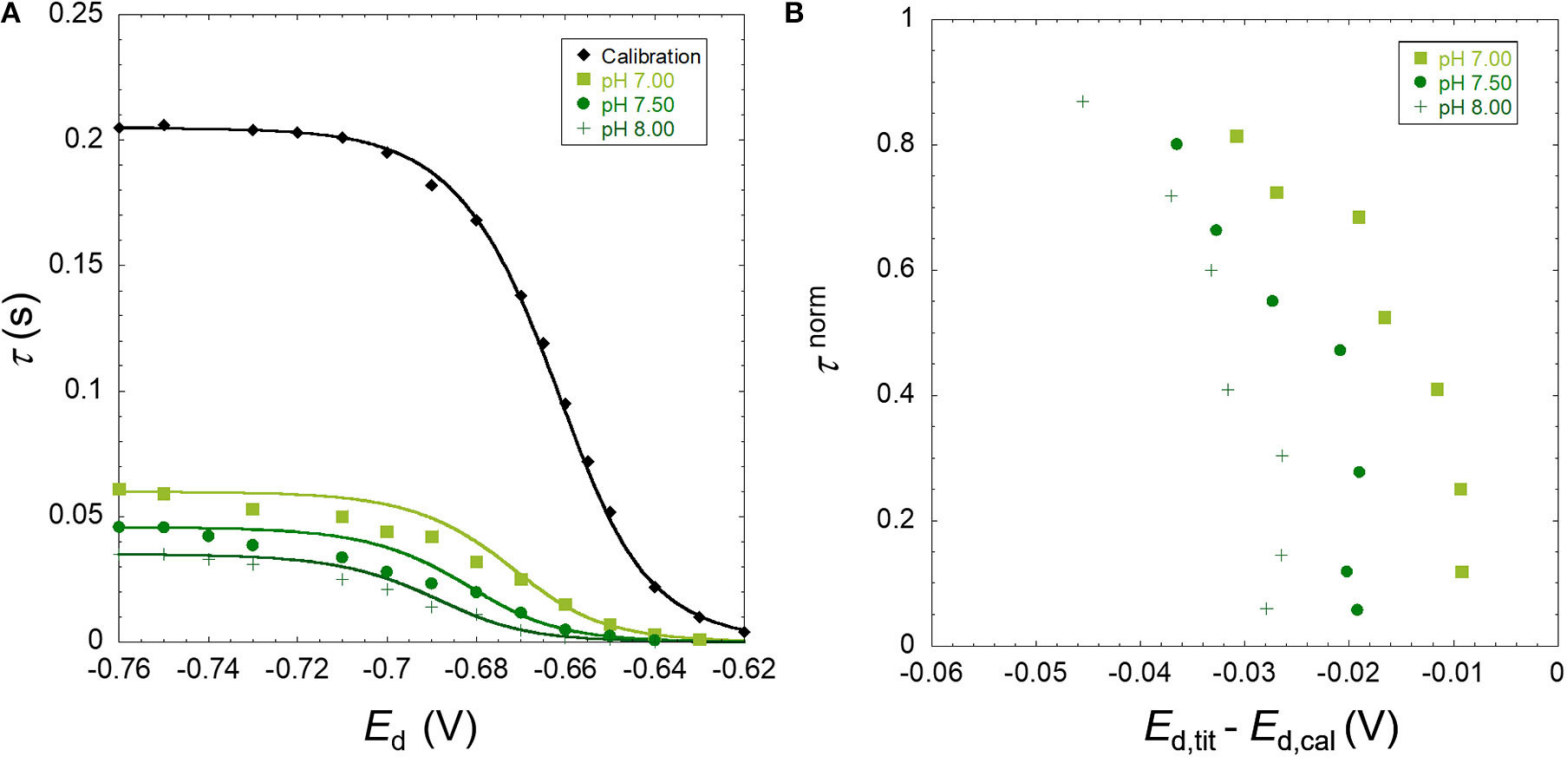
**(A)** Experimental SSCP waves for the cadmium only at pH 4 (calibration) and in the presence of SiO_2_ nanoparticles (TM40) obtained at 10 mM NaNO_3_, pH 7.00, 7.50, and 8.00 for a total Cd concentration of 5 × 10^−4^ mM and particle concentration of 0.3 wt%. The lines represent the analytical simulation of the experimental results as described in the theoretical section. **(B)** The normalized analytical times τ^norm^ derived from the SSCP waves plotted against *E*_d,tit_–*E*_d,cal_.

**Figure 3:**
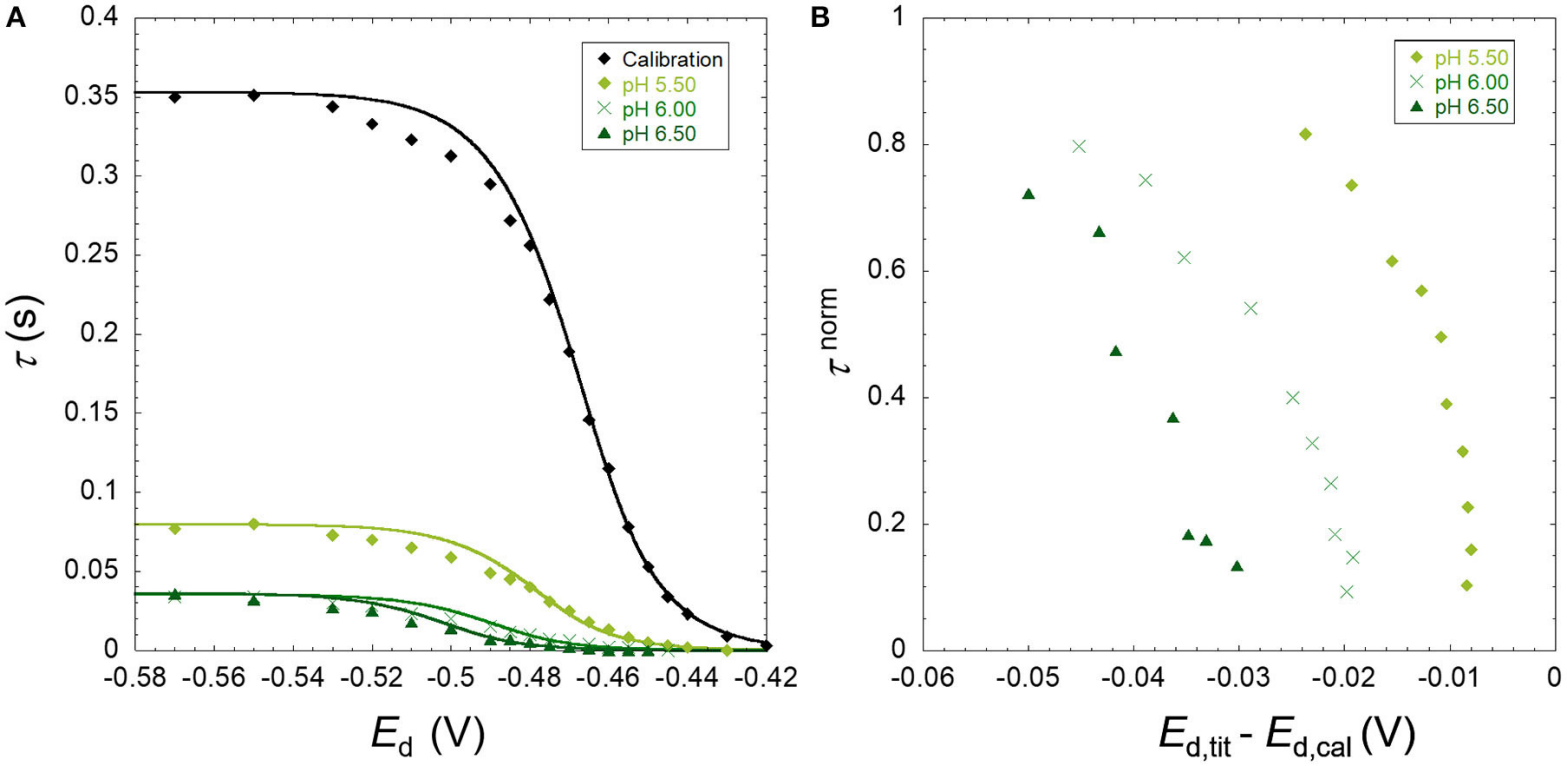
**(A)** Experimental SSCP waves for the lead only at pH 4 (calibration) and in the presence of SiO_2_ nanoparticles (TM40) obtained at 10 mM NaNO_3_, pH 5.50, 6.00, and 6.50 for a total Pb concentration of 5 × 10^−4^ mM and particle concentration of 0.3 wt%. The lines represent the analytical simulation of the experimental results as described in the theoretical section. **(B)** The normalized analytical times τ^norm^ derived from the SSCP waves plotted against *E*_d,tit_–*E*_d,cal_.

The results obtained are presented as raw SSCP waves (A; τ vs. *E*_d_) and double normalized curves (B; τ^norm^ vs *E*_d,tit_-*E*_d,cal_). Each figure contains the information corresponding to a daily experiment comprising a pH titration (three different pH values) at a fixed ionic strength for a metal ion in the presence of nanoparticles. This originates 18 figures corresponding to three metals, three ionic strengths, and two particle sizes. Figures 1–3 presented in this section show the results for Zn, Cd, and Pb in the presence of the TM40 nanoparticle at 10 mM NaNO_3_, whereas the other 15 double normalized curves can be found in Supplementary Figure 1.

As described in the theoretical section, the heterogeneity of the metal binding system modifies the slope of the experimental SSCP waves (left side of Figures 1–3) as compared with the analytical simulation of the homogeneous case (lines) based on the equilibrium bulk situation measured by AGNES. In Figure 3A, a deviation can be observed between the experimental calibration points and the theoretically simulated line. In over 18 experiments performed, we have seen these phenomena only twice; thus, the theoretical simulations were used in our calculations. Similar deviations have been described for pseudopolarograms by Omanović and Branica (2003); hence, a correction reported therein is necessary if a majority of the calibrations present this shape.

The B panels of the three figures give a double normalized representation of the impact of metal binding heterogeneity on SSCP curves as explained above. A homogeneous case is represented by a perfectly vertical alignment, whereas a heterogeneous situation is materialized by a progressive shifting along the y-axis. An increase of the shift in the x-axis with pH indicates a greater binding strength (*K*′) along the lines of the DeFord–Hume theory (DeFord and Hume, 1951).

In some situations, such as the pH 5.5 Pb case, and several other examples in Supplementary Figure 1, one observes two parts in the curve, initially showing a homogeneous tendency (vertical), followed by a marked inclination at higher *E*_d,tit_–*E*_d,cal_ values.

The three metal ions tested show a markedly different behavior. For Zn, the metal interaction with SiO_2_ can be considered as quite homogeneous, with a minor deviation as compared with the fully homogeneous case. In contrast, the lead binding to silica nanoparticle shows a significant heterogeneity, whereas cadmium case corresponds to a slightly heterogeneous system with abundant examples of the two-part curves.

### Impact of pH and Ionic Strengths on the Heterogeneity

The shifting operating at the x-axis upon increase of pH or decrease of ionic strength is due to the higher binding affinity of the metal for the silica particle owing to the increased number of binding sites by deprotonation or the lower screening effect of the electrolyte, respectively. The expected increase of binding strength with pH is observed for all metal cases. In the case of Pb, the heterogeneity degree also increases with pH (from 5.5 to 6.5) as depicted in Figure 3B for Ludox TM40 and Supplementary Figure 1 for Ludox LS30 at 10 mM ionic strength. This pH dependency of the heterogeneity is still observed although less evident for the higher ionic strengths. For Cd (Figure 2B and Supplementary Figure 1) this increase in heterogeneity with pH cannot be observed clearly in the double normalized figures, probably due to experimental errors in the measurement coupled with the smaller heterogeneity evidenced by these cations. Since Zn shows an almost homogeneous behavior, it was not expected to observe significant variations of heterogeneity with pH for this metal. Increasing the ionic strength does not affect the concentration of deprotonated groups in the nanoparticles, but: (i) it does provide a greater screening effect of the particle charges, thus decreasing the electrostatic ion accumulation, and (ii) it impacts the activity coefficients of both ligand and metal ions, thus reducing the covalent binding.

### Influence of the Particle Size on the Heterogeneity

The Ludox LS30 and TM40 nanoparticles are chemically identical, being only different in their physical characteristics as presented in Table 1 (supplier information).

**Table 1:** Physical characteristics of the Ludox nanoparticles.

**Particle**	**Radius (nm)**	**Surface area (BET; m^**2**^ g^**−1**^)**	**Density (g cm^**3**^)**	**Particle number (particle g^**−1**^)**
LS30	8	215	1.2	3.86 × 10^17^
TM40	17	140	1.3	3.74 × 10^16^

In this section, we analyze only the Pb results since this is the metal ion that presents the highest binding heterogeneity. **Figure 5** shows the double normalized curves at 10 mM ionic strength for both nanoparticles at pH 5.5, 6.0, and 6.5. We can observe that there is a difference in the x-axis at the low τ^norm^ where the TM40 values are more negative than the LS30 ones. This indicates a higher TM40 binding strength in the bulk, which is confirmed by the stability constant values presented in Table 2.

**Table 2:** Deprotonated ligand concentrations for the Ludox nanoparticles at 10 mM ionic strength and respective bulk Pb thermodynamic binding constants computed from AGNES results.

	**Ludox LS30**			**Ludox TM40**		
**[SiO_**2**_] (w/w)**	**0.10%**			**0.30%**		
pH	5.50	6.00	6.50	5.50	6.00	6.50
*c*_L, T_ (mM)*	0.013	0.020	0.030	0.037	0.052	0.070
*K*${}_{\text{bulk}}^{\prime }$	2.25	5.78	15.92	8.78	52.81	146.8
log *K*${}_{\text{bulk}}^{**}$	2.25	2.45	2.72	2.38	3.01	3.32

* *Values for Ludox LS30 are from Goveia et al. (2011), and values for TM40 are our own unpublished results*.

** *K_bulk_ in mM equivalent to mol m^−3^*.

At pH 5.5, results suggest that the LS30 is more heterogeneous than the TM40; however, for the higher pH values, this representation does not show meaningful differences. The double normalized representation is not able to discriminate between the Pb/Ludox particle binding heterogeneities; hence, it is necessary to start using the more quantitative full wave analysis to achieve a better discrimination. **Figure 6** shows the result of this analysis for the same points presented in **Figure 5**. One of the best ways to investigate heterogeneity from the full wave analysis results is to draw the electrode surface stability constant *K*^0′^ ($=c_{ML}^{0}$/$c_{M}^{0}$) computed from Equations (9, 10) as function of the total metal surface concentration, $c_{M,T}^{0}$, and compare this with the bulk equilibrium parameter (*K*${}_{\text{bulk}}^{\prime }$). A homogeneous system will have a constant *K*′, whether in the surface or bulk, whereas a heterogeneous system will show a *K*^0′^ decrease with increasing $c_{\text{M},\text{T}}^{0}$.

**Figure 6A** (TM40 and LS30) depicts a situation where both systems have a pronounced heterogeneity. Since they also have quite different bulk stability constants, it is interesting to normalize these curves by their respective *K*${}_{\text{bulk}}^{\prime }$ (**Figure 6B**). By doing so, we observe that the relative heterogeneity of the two systems is clearly different. Pb/TM40 presents a quasi-homogenous behavior between 5 and 3 × 10^−4^ mM, and below this value, it becomes strongly heterogeneous, whereas Pb/LS30 depicts a strong relative heterogeneity in all the concentration range, albeit with significantly more experimental point scattering.

## Discussion

### Methodological Aspects of Heterogeneity Analysis

One of the main advantages of electroanalytical techniques, namely, SSCP, is the ability to scan the low surface coverages that are relevant in environmental systems, where the ligands are usually in excess over the metal ions. In this work, the highest degree of coverage is 4% of deprotonated sites for Pb in the presence of LS30 at pH 5.5- and 10-mM ionic strengths.

**Figure 4:**
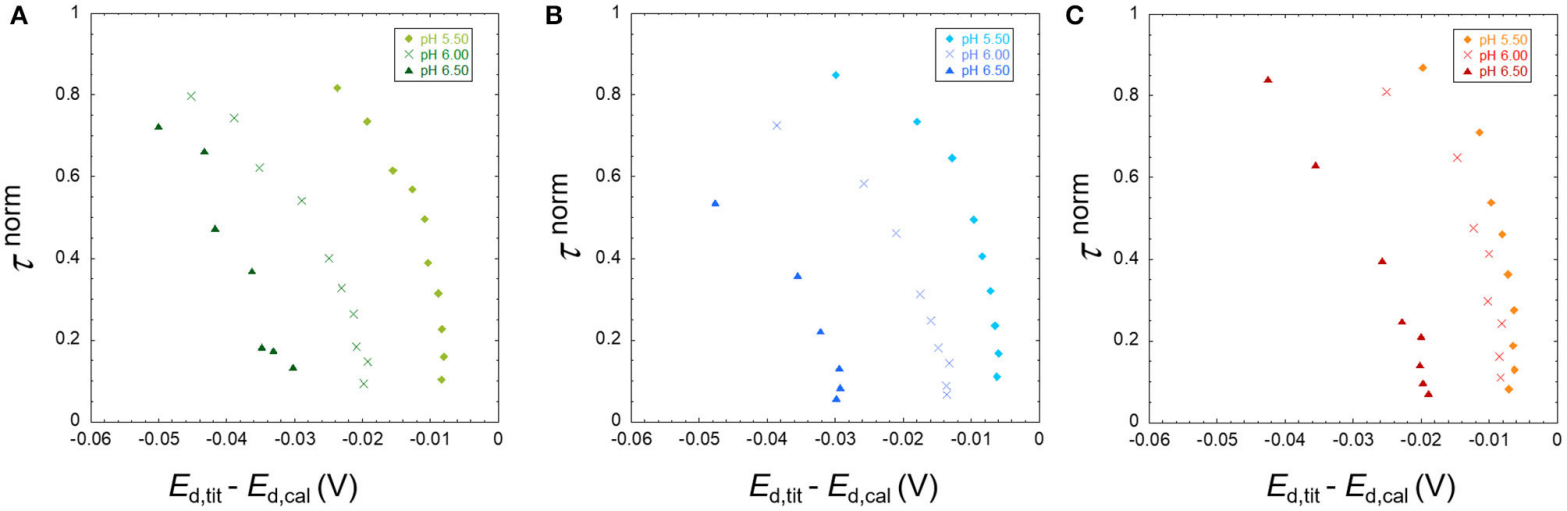
The normalized analytical times τ^norm^ derived from the SSCP waves of Pb in the presence of TM40 silica nanoparticles plotted against *E*_d,tit_–*E*_d,cal_ for the three pH 5.50, 6.00, and 6.50 for a total Pb concentration of 5 × 10^−4^ mM and particle concentration of 0.3 wt%, at ionic strengths of 10 **(A)**, 30 **(B)**, and 100 mM **(C)**.

**Figure 5:**
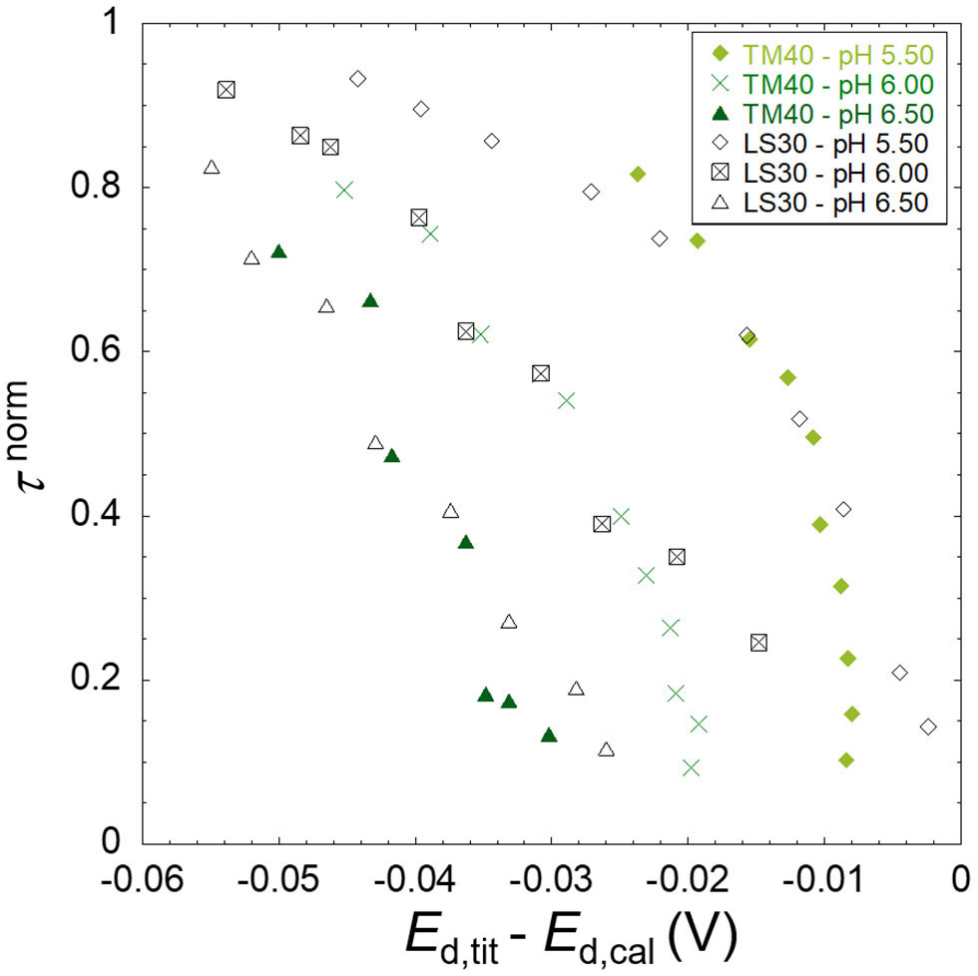
Comparison between the normalized transition times τ^norm^ derived from the SSCP waves of 5 × 10^−4^ mM Pb in the presence of TM40 (0.3 wt%) and LS30 (0.1 wt%) SiO_2_ nanoparticles plotted against *E*_d,tit_–*E*_d,cal_ for the three pH 5.50, 6.00, and 6.50 at ionic strength of 10 mM.

A fast and informative way of obtaining direct qualitative information at low surface coverage on the system heterogeneity is to apply the double normalization of the SSCP wave (Figures 1–5). However, like the Γ parameter of the Freundlich isotherm, this normalized description does not discriminate the electrostatic and chemical binding contributions.

**Figure 6:**
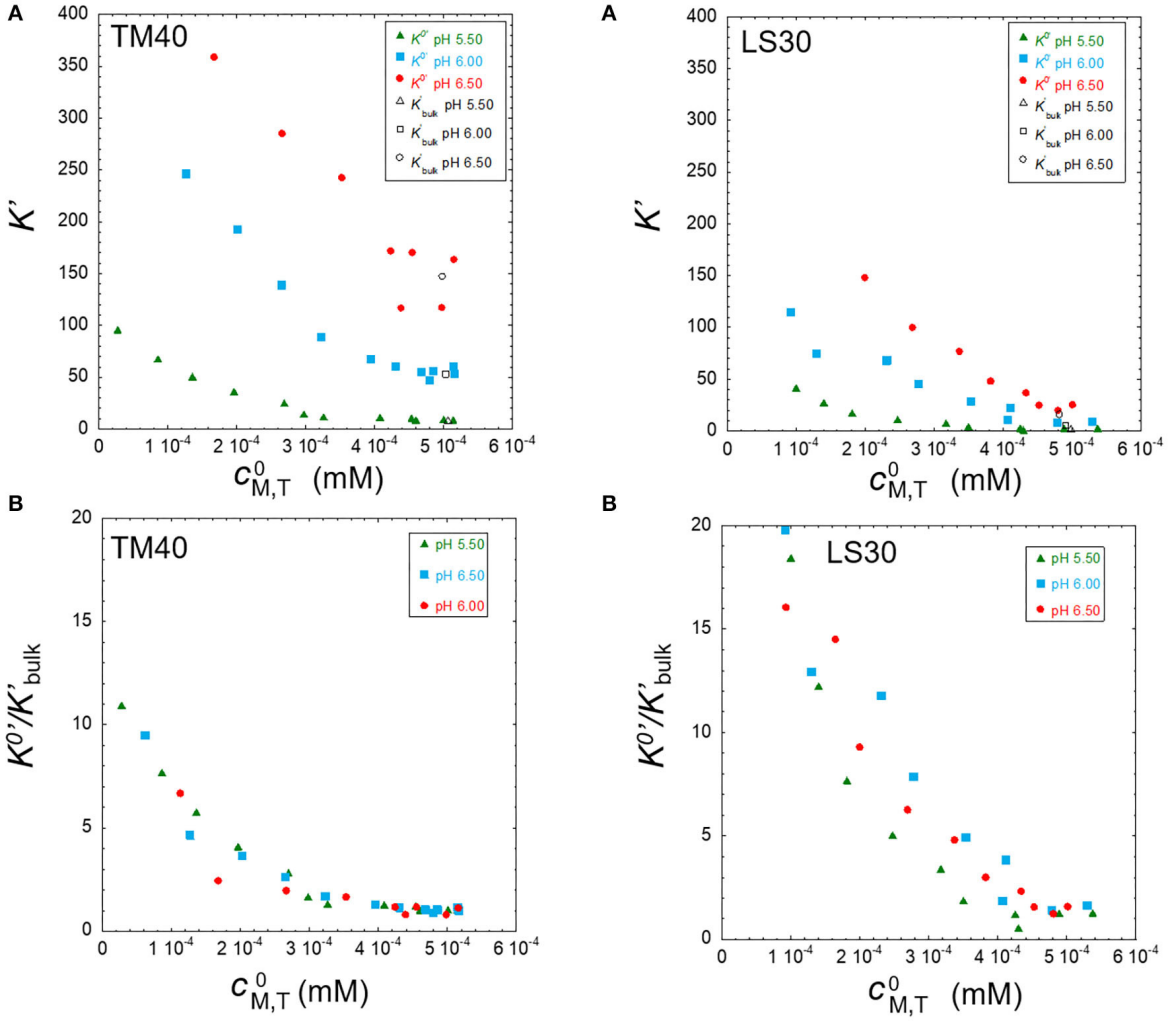
Stability constants at the electrode surface (*K*^0′^) and bulk solution (*K*${}_{\text{bulk}}^{\prime }$) **(A)** and *K*^0′^ normalized by *K*${}_{\text{bulk}}^{\prime }$ **(B)** as function of surface total metal concentration for 5 × 10^−4^ mM Pb in the presence of TM40 (0.3 wt%) and LS30 (0.1 wt%) silica particles for the three pH 5.50, 6.00, and 6.50 at ionic strength of 10 mM.

This qualitative approach can be improved by applying a quantitative full SSCP wave analysis that allows a better interpretation of the experimental data as exemplified by the study of the effect of particle size on the heterogeneity in Figure 6 than the qualitative equivalent in Figure 5.

To advance further into the differentiation of the electrostatic and covalent contributions, it is necessary to interpret the experimental data by fitting the results with pertinent physicochemical models. In a future work, our goal will be to obtain the electrostatic properties of the nanoparticle from protolytic titrations at different ionic strengths. Hence, to determine the electrostatic descriptors, an electrostatic model will be used, namely, the one recently proposed by our group (Pinheiro et al., 2020b). This model applies a Poisson–Boltzmann equation considering particles with an impermeable core and a permeable shell, thus taking into account the permeable gel-like layer present in the SiO_2_/solution interphase (Allison, 2009). Then, the covalent part of the binding will be initially described using a surface complexation model comprising the contribution of mono and bidentate silanol groups and, if necessary, a contribution of stronger aluminum hydroxide groups originating from the aluminum impurity present in the SiO_2_ nanoparticles (Bergna and Roberts, 2005).

### Evaluation of the Heterogeneity for the Different Metal Ions

There is a striking difference between the Pb (Figure 3) and Zn (Figure 1) binding heterogeneities with the SiO_2_ nanoparticles. Pb shows a significant heterogeneity in all conditions, whereas Zn is almost homogeneous at lower pH and higher ionic strengths, showing a small heterogeneity at higher pH and lower ionic strengths.

The Pb heterogeneity starts at lower pH, already evident at pH 5.5, where there are less deprotonated groups (*c*_L, T_) available for covalent binding (Table 2) as well as a smaller electrostatic potential in the nanoparticle. No binding of Zn and Cd was observed in at this pH during the preliminary experiments.

One possible contribution to the Pb heterogeneity is given by the formation of bidentate complexes with the surface silanol groups. Schindler et al. (1976) reported values of log β_2_ of −17.23 and log *K*_1_ of −7.75 for the bidentate and monodentate complexes of Pb, whereas for Cd, only the monodentate complex is formed presenting a log *K*_1_ of −10.4, all values being measured at *I* = 1,000 mM.

The binding heterogeneity of the SiO_2_ nanoparticles is closer to the one observed in environmental mineral particles, such as clays or iron oxyhydroxides, than the strong chemical heterogeneity characteristic of the natural organic matter. In the case of metal association to clay minerals as reported by Rotureau (2014), cadmium displays homogeneous binding dominated by the formation of ion-pair complexes, whereas lead shows a relatively weak chemical heterogeneity, suggesting the formation of edge inner-sphere surface complexes.

Metal/humic matter systems are heterogeneous in nature due to the variety of chemical binding sites present in these colloids, resulting in values of Γ of 0.9–0.8 for Cd and 0.7–0.5 for Pb (Town et al., 2019) depending on the type of organic matter (fulvic, humic, NOM, etc.). In the double normalized curves, this would produce well-spread points with a linear dependency in the x-axis without vertical portions. The mixed curves observed for most of the Cd and some Pb samples are peculiar since it evidences a system that is heterogeneous for low metal-to-ligand ratios and becomes homogeneous for higher degrees of coverage.

Considering the chemically homogeneous nature of the particles, the experimental evidence presented suggests that Pb binding is predominantly covalent, the Zn binding is predominantly electrostatic (low heterogeneity), and the Cd binding is a combination of the two. To quantitatively clarify this aspect, we will carry out the modeling studies described in the previous section on the data presented in this work as future work.

### Evaluation of the Heterogeneity for the Different Nanoparticle Sizes

When comparing the metal binding properties of nanoparticles of different sizes, the key aspect is to consider all the factors that may influence the complexation. In this case, one must consider mass particle concentration, the number of particles, their specific surface area, the permeable shell volume, and the total concentration of deprotonated binding groups as function of pH, as well as the associated surface and shell volume charge densities and potential profile in the shell volume.

Table 3 shows the values for both silica nanoparticles in the measured solutions computed using the manufacturer data. For the LS30 shell volume, we used the values given by Allison (2009) of 9 nm effective particle radius and 1.7 nm shell thickness computed at 10 mM ionic strength. In the absence of measured data for the TM40, we assumed, as a first order approximation, the same increase in radius (+1 nm) and the same shell thickness, i.e., 18 nm radius and 1.7 nm shell thickness.

**Table 3:** Comparison of nanoparticles properties in the measured solution.

	**Ludox LS30**			**Ludox TM40**		
[SiO_2_] (w/w)	0.10%			0.30%		
Particle concentration (particle m^−3^)	4.28 × 10^20^			1.15 × 10^20^		
Surface area (m^2^ m^−3^)	2.38 × 10^5^			4.32 × 10^5^		
Shell volume (m^3^ m^−3^)	6.09 × 10^−4^			7.26 × 10^−4^^(1)^		
pH	5.50	6.00	6.50	5.50	6.00	6.50
*c*_L, T_ (mM)*	0.013	0.020	0.030	0.037	0.052	0.070
Surface charge density (mol m^−2^)	5.34 × 10^−8^	8.51 × 10^−8^	12.7 × 10^−8^	8.53 × 10^−8^	12.0 × 10^−8^	16.3 × 10^−8^
Shell volume charge density (mM)	20.87	33.28	49.68	50.80	71.15	97.00

* *Values for Ludox LS30 are from Goveia et al. (2011), and values for TM40 are our own unpublished results*.

(1) *Value computed by analogy with the Ludox LS30*.

The physicochemical parameters presented in Table 3 suggest that both the Pb binding and heterogeneity would be larger for the TM40 than for the LS30. As commented in the results, Figure 6A shows that the bulk stability constants are effectively larger for the TM40; nonetheless, the binding heterogeneity is surprisingly larger in the case of the LS30. The normalization by the bulk stability constant, *K*${}_{\text{bulk}}^{\prime }$, evidenced a homogeneous/heterogeneous behavior for the TM40 and a predominantly heterogeneous character for the LS30.

Therefore, the parameters given in Table 3 do not explain the larger heterogeneity of the LS30. One possible origin of the heterogeneity difference is the likely presence of aluminum impurities (Bergna and Roberts, 2005). Since the ratio area/volume is larger for smaller particles, it is likely that more Al impurities are present in the surface/shell volume of the LS30 than of the TM40. Another hypothesis for the difference in heterogeneity between the particles arises from the non-homogeneous charge distribution. Allison (2009) suggested that 2/3 of the charge lies in the core surface and 1/3 in the shell volume for the LS30. In this work, we approximated the TM40 shell volume by analogy with the smaller particle, which needs to be experimentally verified. On that point, we previously demonstrated the strong influence of the shell structure and ensuing charge profiles on the metal binding heterogeneity with a different core/shell nanoparticle system (Rotureau et al., 2016).

To tackle this new problem, it is necessary to carry out electrokinetic experiments, in addition to the protolytic titrations, of the two nanoparticles to be able to reconstruct the charge distribution between core surface and shell volume as well as the potential profile in the shell/solution interface, as described by Duval et al. (2005) for the humic substances. This procedure will allow us to obtain a proper electrostatic description of these nanoparticles, prior to application of a surface complexation model, as referred in the Methodological Aspects of Heterogeneity Analysis section.

## Data Availability Statement

The original contributions presented in the study are included in the article/Supplementary Material, further inquiries can be directed to the corresponding author/s.

## Author Contributions

ER: conceptualization, methodology, writing—original draft, and review & editing. LR: investigation, data curation, formal analysis, writing—original draft, and writing-review & editing. DG: investigation and writing—review & editing. NA: investigation and writing—review & editing. JP: conceptualization, methodology, formal analysis, data curation, writing—original draft, and review & editing. All authors contributed to the article and approved the submitted version.

## Conflict of Interest

The authors declare that the research was conducted in the absence of any commercial or financial relationships that could be construed as a potential conflict of interest.
