# Supplementary material for: Investigating the Binding Heterogeneity of Trace Metal Cations With SiO2 Nanoparticles Using Full Wave Analysis of Stripping Chronopotentiometry at Scanned Deposition Potential
Source: Front Chem. 2020 Dec 16;8:614574. doi: 10.3389/fchem.2020.614574 (PMC7772237; doi:10.3389/fchem.2020.614574)
Supplement: Supplementary file 1 [file Data_Sheet_1.PDF]

SUPPORTING INFORMATION

Investigating the binding heterogeneity of trace metal cations with SiO_2 nanoparticles using full wave analysis of stripping chronopotentiometry at scanned deposition potential (SSCP).

Elise Rotureau[a], Luciana S. Rocha[b], Danielle Goveia[c], Nuno G. Alves[b], José Paulo Pinheiro[a,*]

[a] Université de Lorraine, CNRS, LIEC, F-54000 Nancy, France

[b] University of Algarve, CIQA, DQF/FCT, 8005-139 Faro, Portugal

[c] Universidade Estadual Paulista (Unesp), Campus de Itapeva, 18409-010, Itapeva-SP, Brasil

Section A: Theoretical description of AGNES-SCP

The free metal ion concentration was determined by the AGNES-SCP according with the procedure developed by (Parat et al., 2011). This method consists of two conceptual stages: the deposition and stripping stages. Along the first stage, the metal ion in solution M^{2+} is reduced to M^0, until a special situation of Nernstian equilibrium and absence of gradients in the concentration profiles is attained (Galceran et al., 2004). This equilibrium is achieved by applying a deposition potential E_1 more negative than the formal potential E^0 of the couple, for a sufficiently time t_1. In the second stage the concentration of the reduced metal M^0 inside the mercury amalgam is measured by SCP, i.e. the time for complete depletion (transition time, τ) can be determined from the evolution of the recorded potential in response to the imposed stripping current I_s. The faradaic charge (Q) can be rigorously computed as:

$$Q = (I_s - I_{ox})\tau \qquad \text{(S1)}$$

where I_s is the stripping current applied during the stripping step and and I_{ox} is he current due to other oxidants (Parat et al., 2011). Under the experimental conditions used in this work, I_s>> I_{ox} and as a result Eq. (S1) is reduced to:

$$Q = I_s \tau \qquad \text{(S2)}$$

The charge Q is used in this work as the response function for AGNES-SCP and it can be related to the free metal concentration, c_{M}^{*} in the bulk of the solution, with a proportionality factor η_Q (Tehrani et al., 2018):

$$Q = \eta_Q c_{\mathrm{M}}^{*} \qquad \text{(S3)}$$

Therefore, the stability constant in the bulk solution, K'_{bulk}, under the condition where the total ligand concentration in the bulk solution is in excess as compared to the total metal concentration is given by:

$$K'_{\mathrm{bulk}} = \frac{c_{\mathrm{ML}}^{*}}{c_{\mathrm{M}}^{*}} \qquad \text{(S4)}$$

with c_{ML}^{*} the bulk concentration of the metal associated to the silica nanoparticle (calculated from the mass balance of the metal in solution).

Section B: Double normalization representations of the experimental SSCP data for the three metallic ions (Zn, Cd and Pb) at different pH and ionic strength

Zn/TM40 0.15% w/w

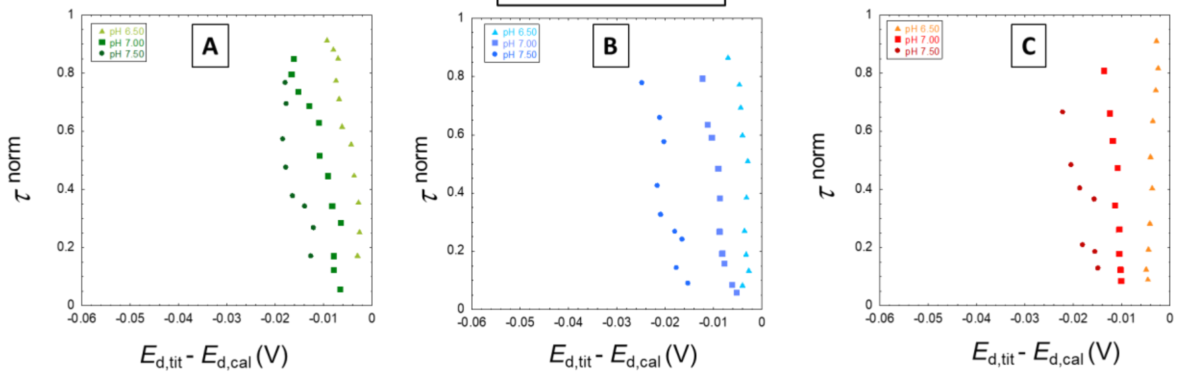

Cd/TM40 0.3% w/w

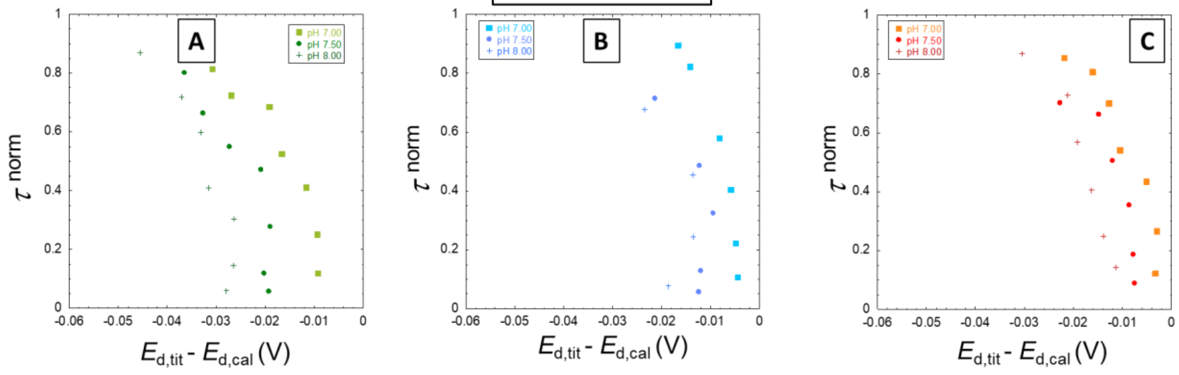

Pb/TM40 0.3% w/w

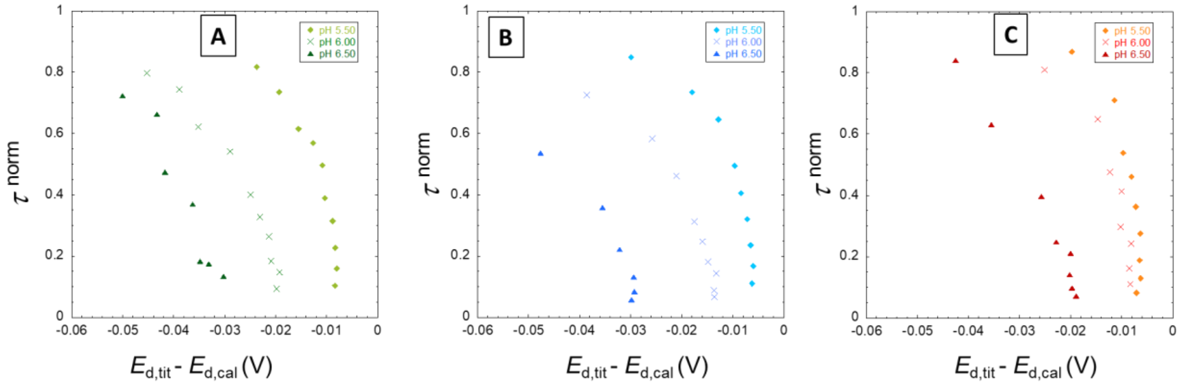

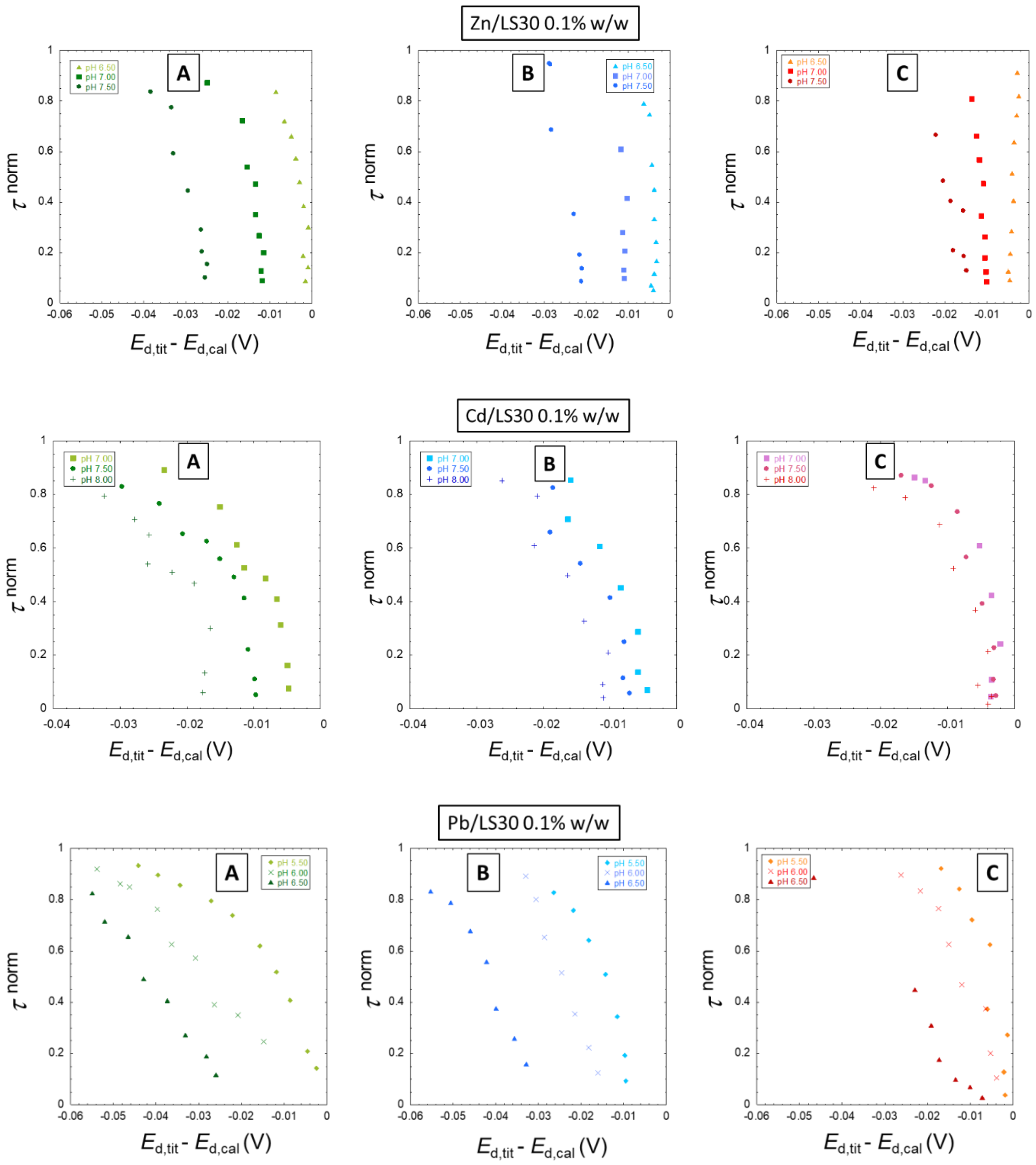

Figure S1: The normalized analytical times τ^{norm} derived from the SSCP waves plotted against $E_{d,tit} - E_{d,cal}$ for the binding between 5x10^{-4} mM Zn, Cd and Pb with Ludox TM40 and Ludox LS30 (concentrations in the figure headers) at the pH given in each legend and ionic strength of 10mM (panels A), 30 mM (panels B) and 100mM (panels C).

References

Galceran, J., Companys, E., Puy, J., Cecilia, J., and Garces, J. L. (2004). AGNES: a new electroanalytical technique for measuring free metal ion concentration. *Journal of Electroanalytical Chemistry* 566, 95–109. doi:10.1016/j.jelechem.2003.11.017.

Parat, C., Schneider, A., Castetbon, A., and Potin-Gautier, M. (2011). Determination of trace metal speciation parameters by using screen-printed electrodes in stripping chronopotentiometry without deaerating. *Anal. Chim. Acta* 688, 156–162. doi:10.1016/j.aca.2010.12.034.

Tehrani, M. H., Companys, E., Dago, A., Puy, J., and Galceran, J. (2018). Free indium concentration determined with AGNES. *Science of The Total Environment* 612, 269–275. doi:10.1016/j.scitotenv.2017.08.200.
